# Single-Cell Sequencing Facilitates Elucidation of HIV Immunopathogenesis: A Review of Current Literature

**DOI:** 10.3389/fimmu.2022.828860

**Published:** 2022-02-03

**Authors:** Silvere D. Zaongo, Vijay Harypursat, Yaokai Chen

**Affiliations:** Division of Infectious Diseases, Chongqing Public Health Medical Center, Chongqing, China

**Keywords:** single-cell sequencing, scRNA-seq, HIV, immunopathogenesis, findings

## Abstract

Knowledge gaps remain in the understanding of HIV disease establishment and progression. Scientists continue to strive in their endeavor to elucidate the precise underlying immunopathogenic mechanisms of HIV-related disease, in order to identify possible preventive and therapeutic targets. A useful tool in the quest to reveal some of the enigmas related to HIV infection and disease is the single-cell sequencing (scRNA-seq) technique. With its proven capacity to elucidate critical processes in cell formation and differentiation, to decipher critical hematopoietic pathways, and to understand the regulatory gene networks that predict immune function, scRNA-seq is further considered to be a potentially useful tool to explore HIV immunopathogenesis. In this article, we provide an overview of single-cell sequencing platforms, before delving into research findings gleaned from the use of single cell sequencing in HIV research, as published in recent literature. Finally, we describe two important avenues of research that we believe should be further investigated using the single-cell sequencing technique.

## Introduction

A broader and deeper knowledge of important immune responses during HIV infection in both the acute and chronic phases of the HIV disease process is likely to assist in the identification of future preventive and therapeutic targets for HIV infection (1). It is well known that cellular immunity is essential for managing infection by intracellular pathogens such as the human immunodeficiency virus (HIV). However, individual cellular dynamics and cell–cell cooperation in developing and coordinating human immune responses are currently insufficiently understood. In this regard, single-cell sequencing represents an excellent alternative to study these processes, as it has evolved into a valuable tool for the understanding of complex multicellular processes in health and disease (2, 3), as well as to expose testable potential therapeutic targets (4). When applied to whole blood as well as a diverse range of human tissues in both healthy and pathological states, single-cell RNA sequencing (scRNA-seq) now allows for the simultaneous study of more than 10,000 single-cell transcriptomes (as suggested by recent improvements to the technique), resulting in the characterization of novel immune cell subsets (5–8). Furthermore, scRNA-seq is now commonly used in immunological studies seeking to describe essential processes in cell formation and differentiation (9, 10), to decipher critical hematopoietic pathways (11–13), and to understand the gene regulatory networks that predict immune function (14–16). A single-cell transcriptome snapshot can yield a valuable insight into the multiple phases of differentiation and activation states that are rarely synchronized between cells. Therefore, the application of scRNA-seq to the HIV research field, and particularly to longitudinal samples, may provide opportunities to discover cellular variables associated with disease progression, without the possibility of confusing these variables with inter-individual variability, as suggested by Martin-Gayo et al. (17). Herein, we briefly review single-cell sequencing platforms before focusing on findings gleaned from the application of single cell sequencing in the HIV field of research, as reported in contemporary literature. Finally, we discuss two critical areas of investigation that we believe are worth exploring by utilizing the single-cell sequencing approach.

## What is Single-Cell Sequencing or Single-Cell RNA Sequencing (scRNA-seq)?

**Figure 1:**
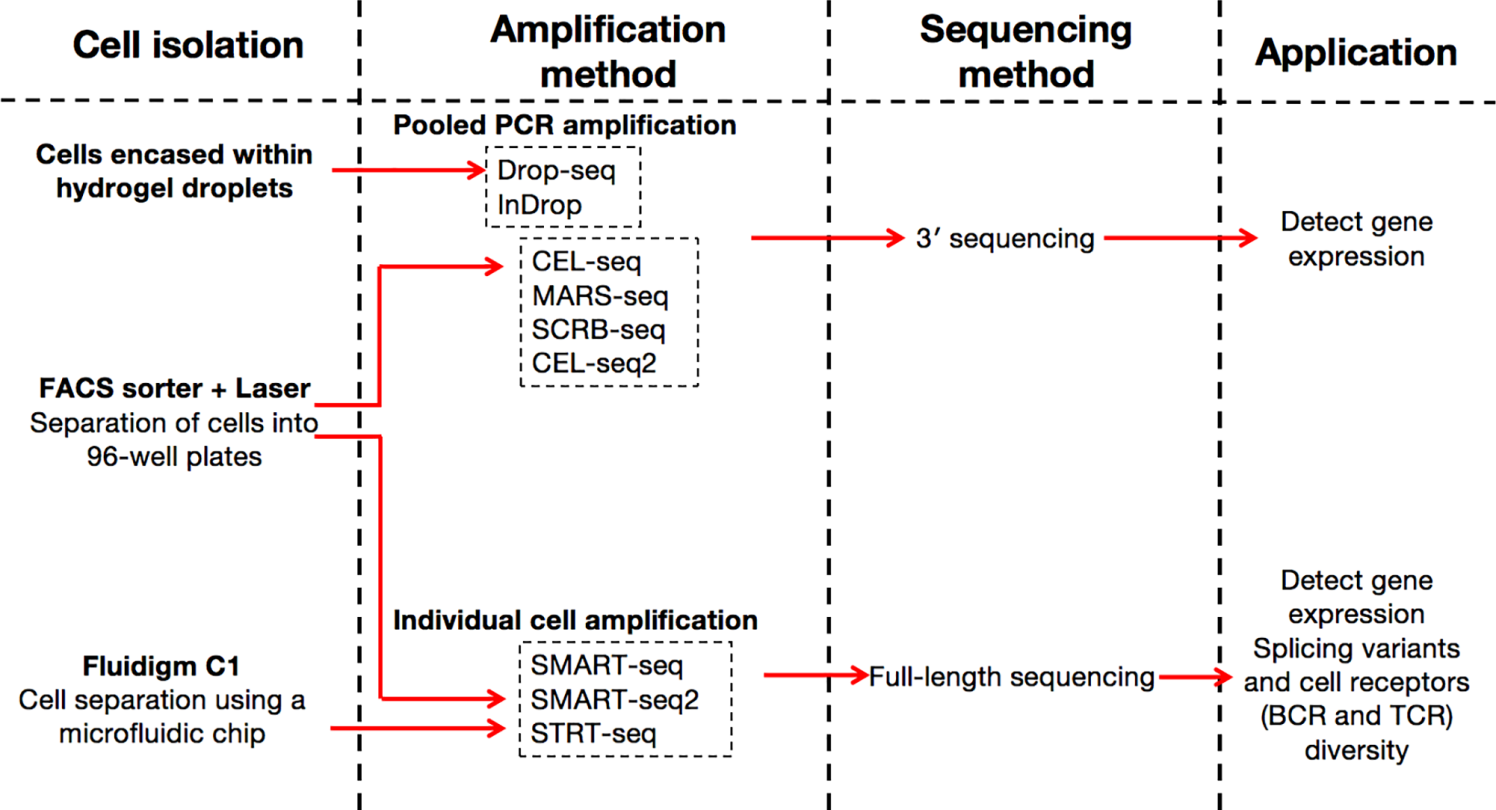
Principle of single-cell sequencing technologies. SCRB-seq, single-cell RNA barcoding and sequencing; BCR, B-cell receptor; TCR, T-cell receptor.

Conventionally, scRNA-seq examines transcripts in a mixture of cells referred to as a ‘bulk’. First proposed in a protocol published in 2009 (18), there are currently many scRNA-seq methods that differ in how the mRNA transcripts are amplified to yield cDNA (full-length or unique molecular identifier) at the 5′ or 3′ end (**Figure 1**). For instance, the switching mechanism at the 5′ end of RNA template sequencing (SMART-seq) (19) and its optimized protocol SMART-seq 2 (20, 21) can generate full-length cDNA. Besides, other methods such as massively parallel RNA single-cell sequencing (MARS-seq) (22), single-cell tagged reverse transcription (STRT) (23, 24), cell expression by linear amplification and sequencing (CEL-seq) (25), CEL- seq2 (26), Drop-seq (6), and indexing droplets (inDrops) (27) are designed to integrate unique molecular identifiers into the cDNA.

Several published articles have already detailed the different methods used for scRNA-seq, their individual advantages, and their limitations (28–30) (**Tables 1**, **2**). However, it is worth noting that to date, despite the existence of several sequencing methods, scRNA-seq remains challenging to conduct since whole-transcriptome amplification methods [SMART-seq, CEL-seq, Quartz-seq (31)] require the processing of hundreds to thousands of single cells, and small volumes of sample (32). Therefore, a number of strategies for the procedure of constructing a scRNA-seq library, such as protocols based on microdroplet technology [i.e., Drop-Seq (6) and DroNc-seq (33)], have been described. These microdroplet and other microwell-based [i.e., microwell-seq (34), Nx1-seq (35) and Seq-Well (5)] protocols make it possible to handle thousands of single cells with ease. Moreover, the currently used method, which combines higher-throughput and lower-cost for scRNA-seq analysis, is the single-cell combinatorial indexing method (sci-RNA-seq) (36, 37). Overall, depending on the platform, issues such as restricted cell capture, low RT efficiency, amplification bias, and the need for a high number of sequencing reads remain. Thus, users should appropriately select methods of scRNA-seq that best suit their specific samples and study objectives.

**Table 1:** Summary of single-cell sequencing applications and methods.

Type	Method	Feature
Transcriptome sequencing	Smart-seq	WTA method; template switching
	CEL-seq	WTA method; *in vitro* transcription
	Quartz-seq	WTA method; poly(A) tagging
	C1-CAGE	5′-end RNA-seq
	RamDa-seq	Total RNA-seq
	Drop-seq	Microdroplet-based method
	Microwell-seq	Microwell-based method
Genome sequencing	MDA	WGA method; isothermal amplification
	DOP-PCR	WGA method; PCR-based
	MALBAC	WGA method; hybrid
Epigenome sequencing	scBS-seq	Whole-genome BS-seq
	scRRBS	RRBS
	scAba-seq	5hmC sequencing
	scATAC-seq	ATAC-seq
	Drop-ChIP	ChIP-seq; microdroplet-based
	scChIC-seq	Ab-Mnase
	CUT&Tag	Ab + protein A-Tn5 transposase
	Single-cell Hi-C	Hi-C
Multilayer sequencing from the same cells	G&T-seq	MDA/PicoPlex (WGA), SMART-seq2 (WTA)
	DR-seq	No physical separation of DNA and RNA
	scM&T-seq	Based on scBS-seq and G&T-seq
	scDam&T-seq	Based on DamID and CEL-seq
	T-ATAC-seq	Based on scATAC-seq and TCR-seq
	SNARE-seq	Tn5-DNA/mRNA captured by beads
	scCAT-seq	Separation of nucleus and cytoplasm
	CITE-seq	Protein detected by barcode-conjugated antibodies
	REAP-seq	Protein detected by barcode-conjugated antibodies

WTA, Whole transcriptome amplification; C1-CAGE, C1-Cap analysis gene expression; RamDa-seq, Random displacement amplification sequencing; WGA, whole-genome amplification; MDA, Multiple displacement amplification; scBS-seq, Single-cell bisulfite sequencing; scRRBS, single-cell reduced-representation bisulfite sequencing; RRBS, Reduced-representation bisulfite sequencing; scAba-seq, Single-cell AbaSI sequencing; scChIC-seq, single-cell chromatin immunocleavage sequencing; CUT&Tag, Cleavage under targets and tagmentation; Ab, antibody; G&T-seq, Genome and transcriptome sequencing; scM&T-seq, Single-cell methylome and transcriptome sequencing; scATAC-seq, Single-cell sequencing assay for transposase-accessible chromatin; T-ATAC-seq, Transcript-indexed ATAC-seq; scCAT-seq, single cell chromatin accessibility and transcriptome sequencing; SNARE-seq, single-nucleus chromatin accessibility and mRNA expression sequencing; CITE-seq, Cellular indexing of transcriptomes and epitopes; REAP-seq, RNA expression and protein sequencing assay.

**Table 2:** Current approaches for scRNA-seq and their practical advantages and limitations.

Available Technologies	Number of Cells/Experiment	Cost ($)	Sensitivity
Plate-based protocols (STRT- seq, SMART-seq, SMART-seq2)	50 to 500	3–6/well	- 7,000 to 10,000 genes per cell for cell lines
			- 2,000 to 6,000 genes per cell for primary cells
Fluidigm C1	48 to 96	35/cell	- 6,000 to 9,000 genes per cell for cell lines
			- 1,000 to 5,000 genes per cell for primary cells
Pooled approaches (CEL-seq, MARS- seq, SCRB-seq, CEL-seq2)	500 to 2,000	3–6/well	- 7,000 to 10,000 genes per cell for cell lines
			- 2,000 to 6,000 genes per cell for primary cells
Massively parallel approaches (Drop-seq, InDrop)	5,000 to 10,000	0.05/cell	- 5,000 genes per cell for cell lines
			- 1,000 to 3,000 genes per cell for primary cells
qPCR	300 to 1,000	1/cell	10 to 30 genes per cell
CyTOF	Millions	35/cell	Up to 40 markers
FACS	Millions	0.05/cell	Up to 17 markers

CyTOF, Cytometry by time of flight; FACS, Fluorescence-activated cell sorting; qPCR, quantitative PCR.

Recently, Kashima et al., have indicated in an extensive review that single-cell sequencing is a formidable tool which has several applications with respect to understanding genetic heterogeneity, detecting footprints of differentiation of individual cells, analysis of the proteome at the single-cell level, integration of different layers of single-cell data sets, and analysis of multilayered sequencing from the same cells (32). In the next section, we review the major findings made by several research groups using single-cell sequencing in HIV research.

## Findings From Single-Cell Sequencing Applications in HIV Research

### Characterization of HIV Replication Cycle

Single-cell sequencing approaches have opened up new avenues of investigation in HIV research. In 2015, a research team provided substantial information on the characterization of HIV replication cycle delays in individual cells (38). Indeed, Holmes et al., found that approximately three hours are required between the onset of early and late HIV-1 gene expression, while matrix protein (MA) causes an approximately 6–12h delay in the generation of extracellular virions. These researchers noted that the delays occur at a time at which apolipoprotein B mRNA editing enzyme catalytic polypeptide-like 3G [APOBEC3G, a molecule which exerts innate antiretroviral immune activity against retroviruses (39, 40)], has largely been removed from the cell, thus suggesting a need to prepare the cells to be efficient producers of infectious HIV-1 virions. Furthermore, Holmes et al., have reported that minor changes (APOBEC3G downregulation, the expression of Gag, the absence of the MA globular head, and the rate at which virions are assembled and released) in the lifespan of infected cells may largely influence viral replication in a single cycle and the overall clinical course in infected individuals, as a typical infected cell generates new virions for only a few hours at the end of a 48h lifespan.

### Identification of New Cell Subsets

Several publications have revealed the ability of scRNA-seq to investigate the complexity and heterogeneity of cell populations during HIV infection. The technique is able to identify the major peripheral blood mononuclear cells (PBMCs) and T-cell subsets affected by HIV infection. For instance, in their study [including 4 healthy donors, 3 donors with a low viral load (LL-HIV), and 3 donors with a high viral load (HL-HIV)], Wang et al. (41),, have identified nine major immune cell clusters, namely CD4^+^ T-cells (CD3D^+^ CD8A^−^ IL7R^hi^), CD8^+^ T-cells (CD3D^+^ CD8A^+^), natural killer cells (NK; CD3D− CD8A^−^ IL7R^−^ GNLY^hi^), B-cells (MS4A1^+^), CD14^+^ monocytes (LYZ^hi^ CD14^hi^), CD16^+^ monocytes (LYZ^hi^ FCGR3A^hi^), conventional dendritic cells (cDCs; LYZ^hi^ FCER1A^hi^), plasmacytoid dendritic cells (pDCs; LYZ^low^ IGJ^hi^), and megakaryocytes (Mk; PPBP^+^). Compared to healthy patients, they noted that CD4^+^ T-cell counts were considerably lower in HL-HIV donors (18.1%, 25.2%, and 3,6% for each of the three HL-HIV donors versus 33.9%, 34%, 53.1%, and 31.1% for each of the healthy donors) while a high proportion of CD4 T-cells was observed in LL-HIV donors (60.7%, 64.3% and 63.1% for each of the three LL-HIV donors). In addition, they identified in the healthy donor PBMCs (i) three CD4 T-cell clusters containing naive CD4^+^ T-cells (CD4-Tn: CD8A^−^ CCR7^+^ IL7R^hi^), effector memory CD4^+^ T-cells (CD4-Tem: CD8A^−^ IL7R^hi^ CCR7^−^ GZMA^+^) (42), and precursor memory cells (CD4-Tpm: CD8A^−^ IL7R^hi^ CCR7^low^ LTB^hi^) and (ii) two CD8^+^ T-cell clusters represented by naive CD8^+^ T-cells (CD8-Tn: CD8A^+^ CCR7^hi^) and effector memory CD8^+^ T-cells (CD8-Tem: CD8A^+^ IL7R^−^ CCR7^−^ GZMA^+^ NKG7^+^). Such a composition of T-cell subtypes was found to be significantly modified in HIV-positive individuals. On the one hand, HL-HIV donors displayed (i) significantly smaller populations of CD4-Tem and CD8-Tn, and (ii) 3 new cell clusters referred to as exhausted memory CD8^+^ T-cells (CD8-Tex), exhausted memory CD4+ T-cells (CD4-Tex), and CD8^+^ Tem cells, with marked upregulation of IFN-response genes (CD8-Tem-IFN^hi^). On the other hand, LL-HIV donors showed (i) a reduction in the CD4-Tem and CD8-Tn clusters, (ii) the appearance of a CD8-Tem-IFN^hi^ cluster, and (iii) the absence of CD4^+^ Tex or CD8^+^ Tex cell populations.

Furthermore, scRNA-seq has been shown to be highly effective in identifying rare (<5% of cells) central nervous system (CNS) immune cell subsets that drive immune activation and neuronal damage during HIV infection. Indeed, Farhadian et al. (43),, by analyzing cerebrospinal fluid (CSF) and blood from adults with and without HIV infection, have identified a rare subset of myeloid cells (microglia-like cells) only present in CSF. Such cells in HIV-positive patients have a particular gene expression signature [overexpression of APOE (Apolipoprotein E), AXL (Tyrosine-protein kinase receptor UFO), CTSB (Cathepsin B), APOC1 (Apolipoprotein C-I), MSR1 (Macrophage scavenger receptor 1), and TREM2 (Triggering receptor expressed on myeloid cells 2)] that matches significantly with neurodegenerative disease-associated microglia (43). With this innovative approach, the preceding authors were able to demonstrate the potential mechanistic link between pathways of neuronal injury in HIV and other neurodegenerative conditions.

In analyzing the PBMCs from four participants who become HIV-positive (untreated) during their study, Kazer et al. (44), reported the presence of (i) well-established PBMC subsets (CD4 T-cells, B-cells, dendritic cells, monocytes, NK cells, cytotoxic T-cells, and plasmablasts), (ii) phenotypic subgroupings of monocytes (antiviral, inflammatory, and nonclassical), (iii) phenotypic subgroupings of cytotoxic T-cells (CTLs, CD8+ CTL), and (iv) NK cell expansion after 2-3 weeks. Interestingly, two patients (P2 and P3), who maintained low levels of viremia (<1000 viral copies/ml) at 2.74 years after infection without ART, exhibited a subset of proliferative cytotoxic NK cells (CD8^-^ TRDC^+^ FCGR3A^+^) during the earliest stages of acute infection. More importantly, this subset of NK cells was found to have increased before the majority of HIV-specific CD8+ T-cells arise.

Overall, scRNA-seq application in the HIV research field is not only continually providing novel information in terms of cell subsets, but also in terms of gene signatures.

### Identification of Exhaustion Signatures

In 2016, Baxter et al. (45),, demonstrated that HIV-infected CD4 T-cells (HIV-infected cells in general) preferentially express markers of exhaustion such as PD-1, CTLA-4, and TIGIT. More specifically, (i) the majority of infected-cells express PD-1, (ii) half of the PD-1^+^ cells also express TIGIT, while TIGIT^+^-only cells are less frequent, and (iii) the frequency of CTLA-4^+^ T-cells was the lowest. These authors were the first to reveal exhaustion signatures during HIV infection through scRNA-seq analysis.

From their observations of the gene signatures of Tex cells in HIV-infected donors (referred to in the preceding section), Wang et al. suggested that CD8-Tex cells show less effector function phenotypes than normal CD8^+^ Tem cells. Indeed, by analyzing the similarities and differences observed in individuals’ (healthy vs. HIV-positive) PBMCs, Wang et al. (41), were able to identify key upregulated genes [killer cell lectin-like receptor subfamily G member 1 (KLRG1), cluster differentiation (CD160), and T-cell immunoreceptor with Ig and ITIM domains (TIGIT)] that are associated with T-cell exhaustion. Interestingly, it appears that KLRG1 blockade effectively restores the function of HIV-specific CD8^+^ T-cells. This finding, possible through scRNA-seq application, highlights the path of a potential target for immunotherapy against HIV infection.

In another study by Nguyen et al. (46),, scRNA-seq was used to investigate the transcriptional signatures of HIV-specific CD8 T-cells present in the lymph nodes (LNs) of elite controllers (ECs) and chronic progressors (CPs). The authors found that the LNs of ECs possess HIV-specific CD8 T-cells displaying lower expression of Perforin-1 (PRF1) and Granzyme B (GZMB) compared to HIV-specific CD8^+^ T-cells from the LNs of CPs. The expression of transcripts for genes encoding for cytolytic molecules, including Granzyme A (GZMA), Granzyme H (GZMH), Granzyme K (GZMK), Granzyme M (GZMM), Fas ligand (FASL), and TNFSF10 [tumor necrosis factor superfamily 10, also known as TRAIL (TNF-related apoptosis-inducing ligand)] (47, 48), was comparable between ECs and CPs, or higher in HIV-specific CD8+ T-cells from the LNs of CPs. Further investigations (flow cytometry, immunohistochemistry, and antibody profiling) have confirmed that in ECs, HIV-specific CD8+ T-cells (i) exhibit weak cytolytic activity, (ii) are present in LN follicles, and (iii) potently suppress HIV replication in the LNs. Additionally, Nguyen et al., demonstrated that HIV-specific CD8^+^ T-cells from the LNs of CPs preferentially express TIGIT, lymphocyte-activation gene 3 (LAG3), and CD244 (recognized as inhibitory receptors), KLRG1, and the transcription factor EOMES (Eomesodermin, also known as T-box brain protein 2, Tbr2). Such a profile perfectly describes an exhausted phenotype [as shown in the literature (49–51)] in HIV-specific CD8^+^ T-cells from the LNs of CPs; whereas, HIV-specific CD8^+^ T-cells from the LNs of ECs preferentially express IL7R, which is essential for homeostasis (52). Furthermore, Nguyen et al., have identified 11 transcripts encoding predicted secreted factors that were selectively upregulated in HIV-specific CD8^+^ T-cells from the LNs of ECs. Among those transcripts, they have reported the presence of genes coding for tumor necrosis factor (TNF), chemokine (C-C motif) ligand 5 (CCL5), ribonuclease A family member 1 (RNASE1), and interleukin 32 (IL32), all known for their ability to suppress HIV replication (53–59).

### Identification of the Inducible Latent Cell and Potential Latent Cells

The application of scRNA-seq in HIV research has revealed the heterogeneity in latent and reactivated HIV-1-infected cells (60–62). It has been demonstrated that latently infected CD4+ T-cells (untreated) display two cell clusters (Cluster 1 and Cluster 2) (63). That is important, as these 2 distinct clusters remain despite treatment with (i) SAHA, a less efficient latency reversing agent (LRA) as shown in the literature (64) or (ii) TCR stimulation (65), which works as a potent LRA. HIV transcript levels were consistently higher in cluster 2 than in cluster 1. This led Golumbeanu et al. (63) to suggest that cluster 1 and 2 represent two distinct states, with different impacts on cellular activation potential and HIV reactivation efficiency. As such, they found that the cells in cluster 1 were in a deeper resting state and difficult to activate upon TCR stimulation. On the other hand, cluster 2 harbored cells in a less deep resting state, were more responsive to cellular activation and HIV expression/reactivation. Deeper investigation by Golumbeanu et al. (63), has uncovered 134 differential expression genes differently expressed between the two distinct cell clusters across all three conditions (untreated, SAHA treatment, or TCR stimulation). Compared to cluster 1, 133 genes were upregulated in cluster 2 (except for the Metazoa_SRP gene). Almost half (48.5%) of those genes represented ribosomal proteins, and an analysis of their enrichment pathways corresponded to processes related to the metabolism of RNA and protein, electron transport, RNA splicing, immune system, HIV infection, and translational regulation. This finding, together with the results obtained using the STRING database online resource (66) to analyze the 134 common differently expressed genes, support the hypothesis of Golumbeanu and her colleagues. Indeed, using STRING, the analysis revealed a strongly connected network of functional interactions and enrichment of viral processes, translational regulation, RNA and protein metabolism, as well as cell activation. Most importantly, these 134 differently expressed genes can be used to identify and discriminate the two clusters *in vivo*. In other words, *via* scRNA-seq, it is now possible to identify the proportion of latent HIV-infected cells that can be successfully reactivated with LRAs. In addition, it seems that HIV is preferentially downregulated (i) in cells with a naive (CCR7^+^ CD45RO^-^) or central memory (CCR7^+^ CD45RO^+^) phenotype and (ii) in cells with higher proliferative potential (67). Furthermore, Liu et al., have found that HIV-1-infected cells (isolated from peripheral blood) from virally suppressed individuals upon early latency reversal preferentially display a T_H_ 1 phenotype (62). It is known that (i) CD4^+^ T cells from peripheral blood are polarized toward T_H_ 1 (often 10-fold more compared to other polarizations) (68), and (ii) HIV-1 also infects T_H_ 1 more frequently (and T_H_ 0 and T_H_ 2 at much lower levels) as reported in the literature (69). The preceding contexts could explain the onset of latency after cell infection by HIV, and also the specific cells to target. As is currently known, the latency process may lead to formation of reservoir cells, which make it challenging to cure HIV.

### Characterization of HIV-1 Reservoir Diversity

Before 2018, researchers using single-cell approaches were oriented to the investigation of cellular heterogeneity of the latent reservoir (45, 70), and the assessment of cellular response heterogeneity to latency reversal agents (LRAs) (71). This is understandable, as latent reservoirs represent the greatest challenge to HIV eradication (72), and the application of LRAs to reverse latency is one of the strategies that has been explored to treat patients (65). For example, Baxter et al. (45), found that latent reservoirs (CD4^+^ T-cells) from HIV-untreated individuals were predominantly central/transitional memory (Tcm/tm, CD27^+^ CD45RA^-^) and Tem (CD27^-^ CD45RA^-^) when stimulated with bryostatin [an antineoplastic drug used in clinical cancer trials (73), and also used as an LRA (65)], or not. Tem (and Tcm contributing to a minor degree) also represented the majority of bryostatin-induced cells (90%) when aviremic ART-treated subjects’ reservoir CD4^+^ T-cells were considered. Even more interestingly, they found that in ART-treated subjects, both Tcm/tm and Tem contributed to the persistent reservoir, and that the bryostatin-induced reaction was limited to the Tem compartment. It has been known for a while that central memory cells represent major long-lived viral reservoirs in ART-treated subjects (74), but the preceding study has revealed the role (in terms of proportion) of effector memory T-cells in HIV reservoir composition.

Recently, Sannier et al. (75), have also used scRNA-seq to explore the diversity of the HIV-1 reservoir. To this purpose, they have considered the active viral reservoir of CD4+ T cells (i) isolated from PBMCs of 16 ART treated and 9 untreated PLWHs, then (2) stimulated for 12h with an LRA, phorbol 12-myristate 13-acetate (PMA)/ionomycine. This stimulation of the active reservoir cells with PMA/ionomycine resulted in a 2-fold and 11-fold median increase in HIV viral RNA-positive (vRNA^+^) in untreated and ART treated samples, respectively. Then, in analyzing the links between viral transcription and translation within ART-treated and untreated individuals, the authors reported that most vRNA^+^ cells in untreated samples express p24 protein. In contrast, the expression of p24 was comparatively infrequent among vRNA+ cells with ART, suggesting a repression of p24 translation in induced viral reservoirs. To further understand the mechanism behind this observation, Sannier et al., analyzed gagRNA and nefRNA co-expression in p24^+^ and p24^-^ vRNA^+^ cells, and found eight theoretical subpopulations of viral reservoirs (**Table 3**). Based on the type of sample, and in the absence of LRA stimulation, an overall consistent hierarchy of the different populations has been reported. In untreated samples, they found: p24^+^ gagRNA^+^ nefRNA^+^ (or p24^+^) > p24 gagRNA^+^ nefRNA (or gagRNA^+^) > p24 gagRNA^+^ nefRNA^+^ (or vRNA_DP_) > p24 gagRNA nefRNA^+^ (or nefRNA+). The hierarchy in ART samples was notably different: gagRNA^+^ ~ nefRNA^+^ > vRNA_DP_ > p24^+^ cells. In comparing both profiles, Sannier et al., suggested that the transcription process is suboptimal in induced viral reservoirs. Indeed, gagRNA^+^ cells showed consistent signs of poor transcriptional activity compared with all other vRNA^+^ subpopulations, and the level of gag transcripts, therefore, may represent key limitations for full gene expression. The expression of CD4 surface protein was much more frequent (CD4 ^high^) on gagRNA^+^ cells and strongly downregulated on p24^+^ cells and vRNA_DP_ (to a lesser extent). In addition, most nefRNA+ cells displayed low CD4 levels. Overall, these results indicate a large heterogeneity of the HIV viral reservoir. In the same manner, a near-full-length single-cell vDNA sequencing of induced, transcriptionally active viral reservoirs have identified underlying proviral defects known to abrogate viral replication, such as inversions, hypermutations, large internal deletions, and premature stop codons. The defect leading to frameshift was also investigated [except in nef (76), as it has been reported to be dispensable for virus replication (77)] as well as J packaging motif, and alterations of the major splice donor (MSD) site (76, 78–82). Sannier et al., have, therefore, found that most transcriptionally active cells harbor packaging signal and MSD site mutations, stop codons/frameshift defects, less common internal deletions (though few occurrences of large deletions in viral genomes harbored by p24+ cells were observed), and a few hypermutated or inverted sequences. The resulting proviral clones also display transcriptional and translational heterogeneity, and besides, identical HIV-1 clones can adopt diverse transcriptional and translational states. Most importantly, they have observed that HIV-1 protein translation in the viral reservoir is associated with an effector memory phenotype, as all viral subpopulations predominantly display a memory phenotype (CD45RA^-^).

**Table 3:** Theoretical subpopulations of viral reservoirs as defined by Sannier et al. (75).

Subpopulations	Gene Characteristics				Proportion
	5’exonRNA	gagRNA	nefRNA	p24	
p24^+^ cells	+	+	+	+	Predominant
vRNA_DP_ cells	+	+	+	–	
gagRNA^+^ cells	+	+	–	–	
nefRNA^+^ cells	+	–	+	–	
Marginal cells	+	+	–	+	Absent in ART-treated and minimal in untreated patients
	+	–	+	+	
	+	–	–	+	
Excluded cells	+	–	–	–	

+, present; -, absent.

In general, studies of the HIV reservoir using the scRNA-seq approach reveal that Tem cells, the major component of HIV reservoir cells, should be the main consideration, especially when such strategies as the “Shock and Kill” strategy (83) or the “SECH” technique (65), aiming to cure HIV infection, are used.

### Identification of Pathogenic TRAIL-Expressing Innate Immune Cells During HIV-1 Infection

In their quest to investigate HIV-induced transcriptomic changes in innate immune cells in lymphoid organs, Cheng et al. (84), used the scRNA-seq approach on hCD45^+^hCD3^–^hCD19^–^ human leukocytes isolated from the spleens of humanized NOD/Rag2^–/–^γc^–/–^ (NRG) mice transplanted with human CD34^+^ hematopoietic stem progenitor cells (NRG-hu HSC mice). Briefly, major innate immune cells, including plasmacytoid dendritic cells (pDCs), myeloid dendritic cells (mDCs), macrophages, NK cells, and innate lymphoid cells (ILCs) were discovered, and in each of them, upregulated genes involved in type I IFN inflammatory pathways were found. A most interesting finding is the particular upregulation of the TNF superfamily member 10 (TNFSF10) gene (which encodes TRAIL) in the aforementioned innate immune cells. The percentage of TRAIL increased from 3.5% (in mock mice) to 37% (in HIV-1–infected mice) in pDCs, 21% to 66% in mDCs, 32% to 81% in macrophages, 14% to 38% in NK cells, and 8% to 56% in ILCs. The upregulation of TRAIL was also reported recently in HIV-specific CD8+ T-cells from the LNs of ECs and CPs, in a relatively high proportion (46). It is known that TRAIL is a proapoptotic ligand with an immune effector function to promote the eradication of infected or malignant cells (85). As such, is it possible that TRAIL plays a role in the depletion of CD4+ T-cells during HIV-1 infection? In trying to provide a clear answer to this question, Cheng et al., found that blockade of the TRAIL signaling pathway in NRG-hu HSC mice prevented HIV-1–induced CD4+ T-cell depletion *in vivo*. In CD4 + T-cells from spleens of humanized mice, they have noted that HIV-1 infection upregulates the expression of TRAIL receptor death receptor 5 (DR5) but not death receptor 4 (DR4). They have, therefore, used a soluble form of DR5 fused with human IgG-Fc (sDR5-Ig) that has the potential to prevent TRAIL-induced cell death (86–88). Identification of pathogenic TRAIL-expressing innate immune cells during HIV-1 infection in mice (84) and humans (46) through scRNA-seq represents a potential therapeutic target. However, even if the number of CD4 T-cells in HIV-1–infected mice treated with sDR5-Ig increased, in comparison to the isotype control treatment group, the number of CD4 T-cells remained lower than that in mock mice (84), suggesting that mechanisms other than the TRAIL pathway may also contribute to CD4+ T-cell depletion *in vivo* (89).

### Effect of Methamphetamine on the SIV-Infected Rhesus Monkey Brain

Using scRNA-seq, Miu et al., have demonstrated the effect of methamphetamine on the brains of SIV-infected rhesus monkeys (90). To this purpose, they isolated microglia and brain macrophages from SIV-infected rhesus monkeys treated with (Meth-SIVE derived cells) and without (SIVE derived cells) methamphetamine. Further experiments were then conducted on these samples. Firstly, they noted that monkeys treated with methamphetamine displayed a significantly increased proportion of microglia and macrophages infected by SIV. Compared to SIVE derived cells, known macrophage/microglia marker genes were elevated (AIF1, 2.4 fold; CD68, 1.5 fold) or decreased (CD163, 2.9 fold; STAB1, 3.0 fold; P2RY12, 6.6 fold; CD14, 1.6 fold; GAS6, 3.4 fold; CSF1R, 2 fold) in Meth-SIVE derived cells. These results informed the authors that Meth-SIVE derived microglia/macrophages mainly differ from SIVE derived cells by a decrease in markers of M2 macrophages, and an alteration in the pattern of activation markers. Pathway analysis using ingenuity pathway analysis (IPA) has, therefore, revealed that the SIV-infected cells from monkeys treated with methamphetamine had increased gene encoding functions in cell death pathways and inhibited the brain-derived neurotropic factor pathway. Further investigation revealed that the gene expression patterns in infected cells (with or without methamphetamine) did not cluster separately from uninfected cells (5 similar clusters in each condition). However, clusters within microglia and/or macrophages from methamphetamine-treated animals differed in neuroinflammatory and metabolic pathways from those comprised of cells from untreated animals. Thus, it appears that methamphetamine, in addition to promoting CNS infection by SIV, has a damaging effect on both infected and uninfected microglia and brain macrophages. Although this investigation utilized simian cells, the study highlights the multiple interactions and consequences of SIV, and by extrapolation, HIV infection and drug usage on the brain.

**Figure 2:**
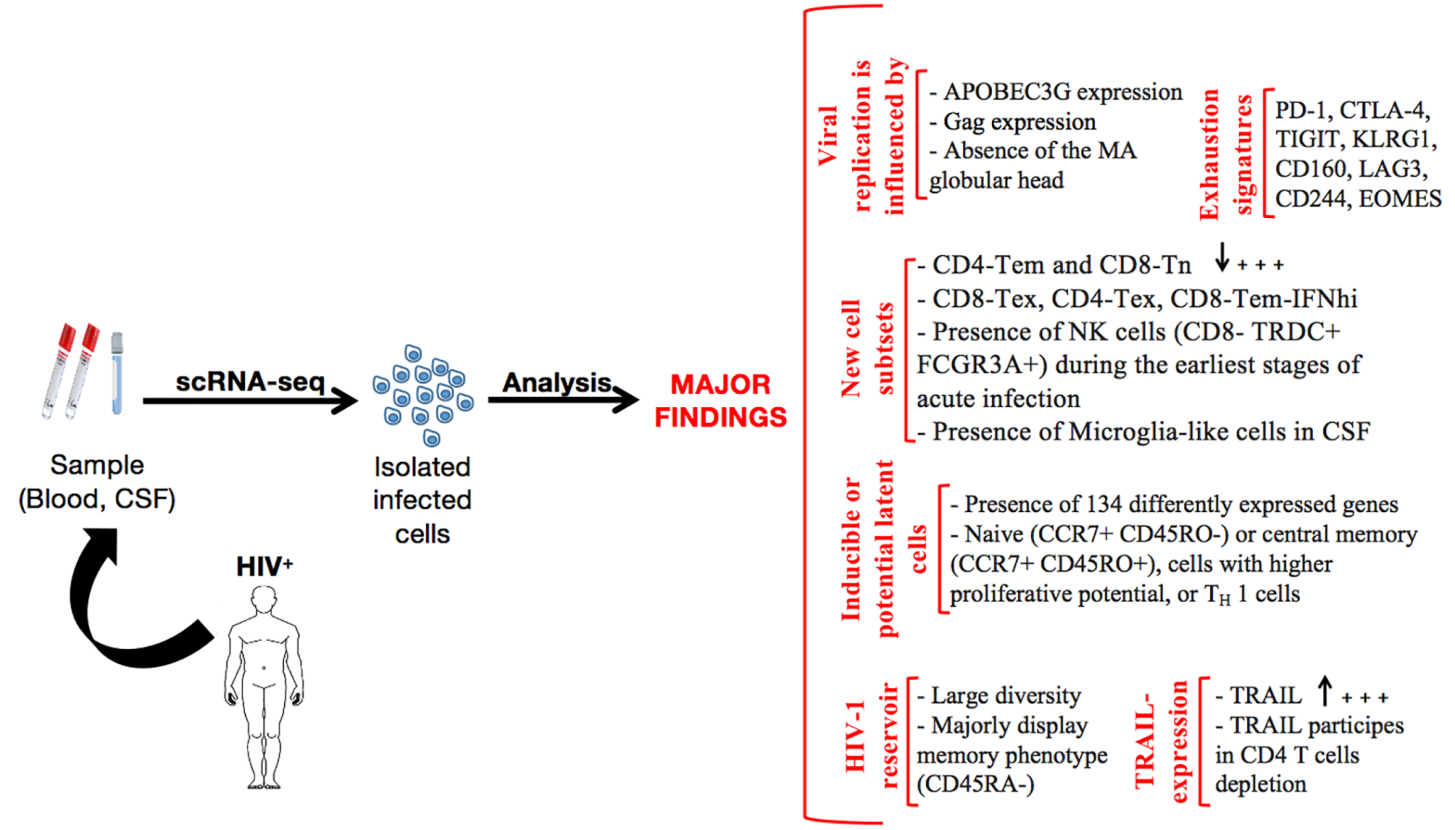
Summary of the major findings resulting from scRNA-seq application in HIV research.

An overall picture of the major findings gleaned from scRNA-seq application during HIV infection is presented in **Figure 2**. However, critical areas, discussed in the following section, are thus far unexplored and should be actively considered in future research work.

## Potential Future Directions

### Elucidate the Underlying Reasons for the Incomplete Immune Recovery Observed in Immunological Non-Responders (INRs)

In 2020, 27.4 million of the 37.6 million people living with HIV (PLWH) were reported to be on ART. This represents more than triple the number of patients on ART recorded in 2010 (7.8 million), and the data also suggests that since 2001, the use of modern ART has prevented 16.2 million deaths (91). Modern ART efficiently suppresses HIV-1 replication by targeting key mechanisms in its life cycle (92). Thus, ART (i) can reduce HIV viral RNA loads to below detectable levels (93, 94), (ii) can increase the circulating number of CD4+ T-cells (95, 96), (iii) can reduce the incidence of AIDS-related disease and/or death (95, 97), and (iv) can effectively prevent the transmission of HIV to uninfected people (98). However, although ART can effectively inhibit HIV replication and reduce HIV-related mortality, 15-30% of individuals, also known as immunological non-responders (INRs), have difficulty in achieving adequate or full immune reconstitution. Indeed, HIV-positive individuals tend not to respond uniformly to ART. Some individuals are able to achieve and maintain undetectable plasma HIV RNA levels, resulting in an increase of CD4+ T-cell counts to >350 cells/mm^3^ (99). These people are referred to as immunological responders (IRs). However, a substantial but poorly described subset of treated people, the INRs, maintain abnormally low peripheral blood CD4+ T-cell counts of <350 cells/mm^3^, or even lower, long after virological suppression has been achieved (100). The reasons for such a disparity in terms of immune recovery remain to be elucidated. Therefore, the application of scRNA-seq in this area of investigation represents a promising method that may lead to potential therapeutic interventions for patients in this situation, especially knowing that this technique has already been effective in finding new cell subtypes, their exhaustion signatures, and the particular heterogeneity of HIV reservoir cells. We believe that its application to the analysis of several types of samples from INRs may reveal critical information. A particular focus on blood, gut, and stool samples should be prioritized, as several studies have shown the close relationship and complex interactions between gut microbes, their metabolites, and the host’s immune system during HIV disease progression (101–106). Besides, deeper profiling of TRAIL and/or other genes upregulated during HIV infection may provide a clearer picture of the mechanisms involved in CD4 depletion and immune recovery in the particular case of INRs.

### Predict the Onset of Opportunistic Infections (OI) During HIV Infection

Opportunistic infections (OIs) occur easily during HIV infection as the immune system of the HIV-infected individual becomes compromised (107). HIV disrupts the immune system, and a weakened immune system makes it increasingly difficult for the body to fight off OIs. Fortunately, ART has drastically limited the onset of OIs in appropriately treated people (108–110). However, the underlying risk of developing OIs in HIV-infected people is always present. Thus, diligent and methodical blood sampling during routine check-ups, followed by scRNA-seq analysis may help to identify critical markers (from cells, transcriptome, or proteome). The idea is to collate the results provided by scRNA-seq analysis before and after the onset of an OI, to compare them, and to thus identify potentially useful biomarkers. The best illustration of this approach is given by Kazer et al., who have compared the results of scRNA-seq of four untreated individuals before and longitudinally during acute HIV infection. In doing so, they were able to conclude that patients who maintained low levels of viremia (<1000 viral copies/ml) at 2.74 years after infection without ART exhibited a subset of proliferative cytotoxic NK cells (CD8^-^ TRDC^+^ FCGR3A^+^) during the earliest stages of acute infection (44). In that particular case, with this subset of proliferative cytotoxic NK cells (CD8^-^ TRDC^+^ FCGR3A^+^), doctors in charge of newly infected patients could be informed of their predicted potential outcomes. We believe that this investigative approach can and should be developed further, and should be aimed towards finding biomarkers that are likely to predict the onset of OIs.

## Conclusion

Single cell RNA sequencing has greatly improved our understanding of HIV immunopathogenesis, especially with respect to its life cycle, the derived-onset of new cell subsets with diverse and/or particular gene signatures, the infected-cell exhaustion profile, and reservoir cell heterogeneity, to list a few. Several points discussed in this article have the potential to possibly lead to the identification of new therapeutic targets. However, two critical problems often encountered during HIV infection remain unexplored with scRNA-seq. Firstly, finding the causes of abnormal/incomplete immune recovery may help INRs to achieve immune recovery, just as IRs do. Secondly, finding markers that predict the possible onset of an OI will greatly help HIV-positive individuals to improve their overall quality of life. Much missing information regarding HIV infection remains to be elucidated; however, we believe that the scRNA-seq approach combined with other powerful assays/approaches (multiplex of transcriptome, genomic, chromatin, methylation, and/or proteomic assays, to list a few) will certainly enhance the quest to reveal some of the enigmas related to HIV infection and disease in humans in the future.

Two major limitations of currently available single cell assays require mentioning. The first is related to the various omics profiles of each individual cell, which are difficult to process (these comprise high-dimensional and mostly sparse data). Since it has been observed that less sampling bias and fewer batch effects are involved in single cell sequencing, multiomics data analysis from a single cell is, therefore, much more reliable than the integration of single omics layers. At the same time, single-layered data from single cells are easier to obtain, and their integration may allow more cost-effective and less time-consuming analysis. The second major limitation of currently available single cell assays is that results obtained using single-cell sequencing technologies lack meaningful spatial information. The reason for this is that specific tissues are dissociated into single cells before sequencing analysis can proceed. Recently, spatial transcriptome techniques have been proposed [Slide-seq and Visium (10× Genomics/Spatial Transcriptomics) approaches]. However, these existing approaches are not currently available at single cell resolution. With the inexorable progress being made in this exciting research field, we are indeed hopeful that these limitations will be overcome in the near future, and that single cell assays will be used more frequently at a population scale to achieve a more comprehensive understanding of complex disease pathogenesis, for example, as in the pathogenesis of HIV-related disease, and not only for identification of cell population in a heterogeneous tissue.

## Author Contributions

SZ wrote the manuscript and conceived the figures. VH and YC revised and provided significant inputs. All authors read the article and approved the submitted version.

## Funding

This work was funded by Chongqing Talent Cultivation Program (cstc2021ycjh-bgzxm0275).

## Conflict of Interest

The authors declare that the research was conducted in the absence of any commercial or financial relationships that could be construed as a potential conflict of interest.

## Publisher’s Note

All claims expressed in this article are solely those of the authors and do not necessarily represent those of their affiliated organizations, or those of the publisher, the editors and the reviewers. Any product that may be evaluated in this article, or claim that may be made by its manufacturer, is not guaranteed or endorsed by the publisher.

## Abbreviations

AIF1, Allograft inflammatory factor 1; APOBEC3G, Apolipoprotein B mRNA editing enzyme catalytic polypeptide-like 3G; APOC1, Apolipoprotein C-I; APOE, Apolipoprotein E; ART, Antiretroviral therapy; AXL, Tyrosine-protein kinase receptor UFO; BCR, B-cell receptor; CCL5, Chemokine (C-C motif) ligand 5; CD4, Cluster of differentiation 4; CD8, Cluster of differentiation 8; CD14, Cluster of differentiation 14; CD68, Cluster of differentiation 68; CD163, Cluster of differentiation 163; CD244, Cluster of differentiation 244; CD4-Tex, Exhausted CD4 T-cell; CD8-Tex, Exhausted CD8 T-cell; CD4-Tem, Effector memory CD4 T-cell; CD4-Tn, Naive CD4 T-cell; CD4-Tpm, Precursor memory cell; CD8-Tem, Effector memory CD8 T-cell; CD8-Tn, Naive CD8 T-cells; cDC, Conventional dendritic cell; cDNA, Complementary DNA; CEL-seq, Cell expression by linear amplification and sequencing; CNS, Central nervous system; CP, Chronic progressor; CSF, Cerebrospinal fluid; CSF1R, Colony stimulating factor 1 receptor; CTL, Cytotoxic T-cell; CTLA-4, also known as CD152 (cluster of differentiation 152); CTSB, Cathepsin B; DNA, Deoxyribonucleic acid; DC, Dendritic cell; EC, Elite controller; EOMES, Eomesodermin; FASL, Fas ligand; Gag, Group-specific antigen; GAS6, Growth arrest – specific 6; GZMA, Granzyme A; GZMB, Granzyme B; GZMH, Granzyme H; GZMK, Granzyme K; GZMM, Granzyme M; HIV-1, Human immunodeficiency virus type 1; IFN, Interferon; IL32, Interleukin 32; ILC, Innate lymphoid cell; inDrops, indexing droplets; INR, Immunological nonresponder; IR, Immunological responder; IPA, Ingenuity pathway analysis; KLRG1, Killer cell lectin-like receptor subfamily G member 1; LAG3, Lymphocyte-activation gene 3; LN, Lymph nodes; LRA, Latency reversing agent; MARS-seq, Massively parallel RNA single-cell sequencing; MA, Matrix protein; mDC, Myeloid dendritic cell; mRNA, Messenger RNA; MSD, Major splice donor; MSR1, Macrophage scavenger receptor 1; Nef, Negative factor; NK cell, Natural killer cell; OI, Opportunistic infection; P2RY12, Purinergic receptor P2Y12; PBMC, Peripheral blood mononuclear cell; PD-1, Programmed cell death protein 1; pDC, Plasmacytoid dendritic cell; PRF1, Perforin-1; PLWH, People living with HIV; RNA, Ribonucleic acid; RNASE1, Ribonuclease A family member 1; SAHA, Suberoylanilide hydroxamic acid; scRNA-seq, Single-cell RNA sequencing; sci-RNA-seq, Single-cell combinatorial indexing method; SECH, Selective elimination of host cells capable of producing HIV; SIV, Simian immunodeficiency virus; SMART-seq, Switching mechanism at 5′ end of RNA template sequencing; STAB1, Stabilin-1; STRT, Single-cell tagged reverse transcription; TCR, T-cell receptor; Tem, Effector memory T-cell; TIGIT, T-cell immunoreceptor with Ig and ITIM domains; TNF, Tumor necrosis factor; TNFSF10, Tumor necrosis factor superfamily 10 also known as TRAIL; TRAIL, TNF-related apoptosis-inducing ligand; TREM2, Triggering receptor expressed on myeloid cells 2; vRNA^+^, HIV viral RNA-positive.

## Glossary

AIF1: Allograft inflammatory factor 1
APOBEC3G: Apolipoprotein B mRNA editing enzyme catalytic polypeptide-like 3G
APOC1: Apolipoprotein C-I
APOE: Apolipoprotein E
ART: Antiretroviral therapy
AXL: Tyrosine-protein kinase receptor UFO
BCR: B-cell receptor
CCL5: Chemokine (C-C motif) ligand 5
CD4: Cluster of differentiation 4
CD8: Cluster of differentiation 8
CD14: Cluster of differentiation 14
CD68: Cluster of differentiation 68
CD163: Cluster of differentiation 163
CD244: Cluster of differentiation 244
CD4-Tex: Exhausted CD4 T-cell
CD8-Tex: Exhausted CD8 T-cell
CD4-Tem: Effector memory CD4 T-cell
CD4-Tn: Naive CD4 T-cell
CD4-Tpm: Precursor memory cell
CD8-Tem: Effector memory CD8 T-cell
CD8-Tn: Naive CD8 T-cells
cDC: Conventional dendritic cell
cDNA: Complementary DNA
CEL-seq: Cell expression by linear amplification and sequencing
CNS: Central nervous system
CP: Chronic progressor
CSF: Cerebrospinal fluid
CSF1R: Colony stimulating factor 1 receptor
CTL: Cytotoxic T-cell
CTLA-4: also known as CD152 (cluster of differentiation 152)
CTSB: Cathepsin B
DNA: Deoxyribonucleic acid
DC: Dendritic cell
EC: Elite controller
EOMES: Eomesodermin
FASL: Fas ligand
Gag: Group-specific antigen
GAS6: Growth arrest – specific 6
GZMA: Granzyme A
GZMB: Granzyme B
GZMH: Granzyme H
GZMK: Granzyme K
GZMM: Granzyme M
HIV-1: Human immunodeficiency virus type 1
IFN: Interferon
IL32: Interleukin 32
ILC: Innate lymphoid cell
inDrops: indexing droplets
INR: Immunological nonresponder
IR: Immunological responder
IPA: Ingenuity pathway analysis
KLRG1: Killer cell lectin-like receptor subfamily G member 1
LAG3: Lymphocyte-activation gene 3
LN: Lymph nodes
LRA: Latency reversing agent
MARS-seq: Massively parallel RNA single-cell sequencing
MA: Matrix protein
mDC: Myeloid dendritic cell
mRNA: Messenger RNA
MSD: Major splice donor
MSR1: Macrophage scavenger receptor 1
Nef: Negative factor
NK cell: Natural killer cell
OI: Opportunistic infection
P2RY12: Purinergic receptor P2Y12
PBMC: Peripheral blood mononuclear cell
PD-1: Programmed cell death protein 1
pDC: Plasmacytoid dendritic cell
PRF1: Perforin-1
PLWH: People living with HIV
RNA: Ribonucleic acid
RNASE1: Ribonuclease A family member 1
SAHA: Suberoylanilide hydroxamic acid
scRNA-seq: Single-cell RNA sequencing
sci-RNA-seq: Single-cell combinatorial indexing method
SECH: Selective elimination of host cells capable of producing HIV
SIV: Simian immunodeficiency virus
SMART-seq: Switching mechanism at 5′ end of RNA template sequencing
STAB1: Stabilin-1
STRT: Single-cell tagged reverse transcription
TCR: T-cell receptor
Tem: Effector memory T-cell
TIGIT: T-cell immunoreceptor with Ig and ITIM domains
TNF: Tumor necrosis factor
TNFSF10: Tumor necrosis factor superfamily 10 also known as TRAIL
TRAIL: TNF-related apoptosis-inducing ligand
TREM2: Triggering receptor expressed on myeloid cells 2
vRNA^+^: HIV viral RNA-positive

## References

[1] Robb ML Ananworanich J. Lessons From Acute HIV Infection. Curr Opin HIV AIDS (2016) 11(6):555–60. doi: 10.1097/coh.0000000000000316 PMC5642316 27716734

[2] Regev A Teichmann SA Lander ES Amit I Benoist C Birney E. The Human Cell Atlas. eLife (2017) 6:e27041. doi: 10.7554/eLife.27041 29206104 PMC5762154

[3] Gomes T Teichmann SA Talavera-López C. Immunology Driven by Large-Scale Single-Cell Sequencing. Trends Immunol (2019) 40(11):1011–21. doi: 10.1016/j.it.2019.09.004 31645299

[4] Shalek AK Benson M. Single-Cell Analyses to Tailor Treatments. Sci Transl Med (2017) 9(408):eaan4730. doi: 10.1126/scitranslmed.aan4730 28931656 PMC5645080

[5] Gierahn TM Wadsworth MH 2nd Hughes TK Bryson BD Butler A Satija R. Seq-Well: Portable, Low-Cost RNA Sequencing of Single Cells at High Throughput. Nat Methods (2017) 14(4):395–8. doi: 10.1038/nmeth.4179 PMC5376227 28192419

[6] Macosko EZ Basu A Satija R Nemesh J Shekhar K Goldman M. Highly Parallel Genome-Wide Expression Profiling of Individual Cells Using Nanoliter Droplets. Cell (2015) 161(5):1202–14. doi: 10.1016/j.cell.2015.05.002 PMC4481139 26000488

[7] Villani AC Satija R Reynolds G Sarkizova S Shekhar K Fletcher J. Single-Cell RNA-Seq Reveals New Types of Human Blood Dendritic Cells, Monocytes, and Progenitors. Science (2017) 356(6335):eaah4573. doi: 10.1126/science.aah4573 28428369 PMC5775029

[8] Zheng GX Terry JM Belgrader P Ryvkin P Bent ZW Wilson R. Massively Parallel Digital Transcriptional Profiling of Single Cells. Nat Commun (2017) 8:14049. doi: 10.1038/ncomms14049 28091601 PMC5241818

[9] Björklund ÅK Forkel M Picelli S Konya V Theorell J Friberg D. The Heterogeneity of Human CD127(+) Innate Lymphoid Cells Revealed by Single-Cell RNA Sequencing. Nat Immunol (2016) 17(4):451–60. doi: 10.1038/ni.3368 26878113

[10] See P Dutertre CA Chen J Günther P McGovern N Irac SE. Mapping the Human DC Lineage Through the Integration of High-Dimensional Techniques. Science (2017) 356(6342):eaag3009. doi: 10.1126/science.aag3009 28473638 PMC7611082

[11] Paul F Arkin Y Giladi A Jaitin DA Kenigsberg E Keren-Shaul H. Transcriptional Heterogeneity and Lineage Commitment in Myeloid Progenitors. Cell (2015) 163(7):1663–77. doi: 10.1016/j.cell.2015.11.013 26627738

[12] Schlitzer A Sivakamasundari V Chen J Sumatoh HR Schreuder J Lum J. Identification of Cdc1- and Cdc2-Committed DC Progenitors Reveals Early Lineage Priming at the Common DC Progenitor Stage in the Bone Marrow. Nat Immunol (2015) 16(7):718–28. doi: 10.1038/ni.3200 26054720

[13] Mass E Ballesteros I Farlik M Halbritter F Günther P Crozet L. Specification of Tissue-Resident Macrophages During Organogenesis. Science (2016) 353(6304):aaf4238. doi: 10.1126/science.aaf4238 27492475 PMC5066309

[14] Dixit A Parnas O Li B Chen J Fulco CP Jerby-Arnon L. Perturb-Seq: Dissecting Molecular Circuits With Scalable Single-Cell RNA Profiling of Pooled Genetic Screens. Cell (2016) 167(7):1853–66.e17. doi: 10.1016/j.cell.2016.11.038 27984732 PMC5181115

[15] Jaitin DA Weiner A Yofe I Lara-Astiaso D Keren-Shaul H David E. Dissecting Immune Circuits by Linking CRISPR-Pooled Screens With Single-Cell RNA-Seq. Cell (2016) 167(7):1883–96.e15. doi: 10.1016/j.cell.2016.11.039 27984734

[16] Shalek AK Satija R Adiconis X Gertner RS Gaublomme JT Raychowdhury R. Single-Cell Transcriptomics Reveals Bimodality in Expression and Splicing in Immune Cells. Nature (2013) 498(7453):236–40. doi: 10.1038/nature12172 PMC3683364 23685454

[17] Martin-Gayo E Cole MB Kolb KE Ouyang Z Cronin J Kazer SW. A Reproducibility-Based Computational Framework Identifies an Inducible, Enhanced Antiviral State in Dendritic Cells From HIV-1 Elite Controllers. Genome Biol (2018) 19(1):10. doi: 10.1186/s13059-017-1385-x 29378643 PMC5789701

[18] Tang F Barbacioru C Wang Y Nordman E Lee C Xu N. mRNA-Seq Whole-Transcriptome Analysis of a Single Cell. Nat Methods (2009) 6(5):377–82. doi: 10.1038/nmeth.1315 19349980

[19] Ramsköld D Luo S Wang YC Li R Deng Q Faridani OR. Full-Length mRNA-Seq From Single-Cell Levels of RNA and Individual Circulating Tumor Cells. Nat Biotechnol (2012) 30(8):777–82. doi: 10.1038/nbt.2282 PMC3467340 22820318

[20] Picelli S Björklund ÅK Faridani OR Sagasser S Winberg G Sandberg R. Smart-Seq2 for Sensitive Full-Length Transcriptome Profiling in Single Cells. Nat Methods (2013) 10(11):1096–8. doi: 10.1038/nmeth.2639 24056875

[21] Picelli S Faridani OR Björklund AK Winberg G Sagasser S Sandberg R. Full-Length RNA-Seq From Single Cells Using Smart-Seq2. Nat Protoc (2014) 9(1):171–81. doi: 10.1038/nprot.2014.006 24385147

[22] Jaitin DA Kenigsberg E Keren-Shaul H Elefant N Paul F Zaretsky I. Massively Parallel Single-Cell RNA-Seq for Marker-Free Decomposition of Tissues Into Cell Types. Science (2014) 343(6172):776–9. doi: 10.1126/science.1247651 PMC4412462 24531970

[23] Islam S Kjällquist U Moliner A Zajac P Fan JB Lönnerberg P. Characterization of the Single-Cell Transcriptional Landscape by Highly Multiplex RNA-Seq. Genome Res (2011) 21(7):1160–7. doi: 10.1101/gr.110882.110 PMC3129258 21543516

[24] Islam S Zeisel A Joost S La Manno G Zajac P Kasper M. Quantitative Single-Cell RNA-Seq With Unique Molecular Identifiers. Nat Methods (2014) 11(2):163–6. doi: 10.1038/nmeth.2772 24363023

[25] Hashimshony T Wagner F Sher N Yanai I. CEL-Seq: Single-Cell RNA-Seq by Multiplexed Linear Amplification. Cell Rep (2012) 2(3):666–73. doi: 10.1016/j.celrep.2012.08.003 22939981

[26] Hashimshony T Senderovich N Avital G Klochendler A de Leeuw Y Anavy L. CEL-Seq2: Sensitive Highly-Multiplexed Single-Cell RNA-Seq. Genome Biol (2016) 17:77. doi: 10.1186/s13059-016-0938-8 27121950 PMC4848782

[27] Klein AM Mazutis L Akartuna I Tallapragada N Veres A Li V. Droplet Barcoding for Single-Cell Transcriptomics Applied to Embryonic Stem Cells. Cell (2015) 161(5):1187–201. doi: 10.1016/j.cell.2015.04.044 PMC4441768 26000487

[28] Giladi A Amit I. Single-Cell Genomics: A Stepping Stone for Future Immunology Discoveries. Cell (2018) 172(1-2):14–21. doi: 10.1016/j.cell.2017.11.011 29328909

[29] Papalexi E Satija R. Single-Cell RNA Sequencing to Explore Immune Cell Heterogeneity. Nat Rev Immunol (2018) 18(1):35–45. doi: 10.1038/nri.2017.76 28787399

[30] Hedlund E Deng Q. Single-Cell RNA Sequencing: Technical Advancements and Biological Applications. Mol Aspects Med (2018) 59:36–46. doi: 10.1016/j.mam.2017.07.003 28754496

[31] Sasagawa Y Nikaido I Hayashi T Danno H Uno KD Imai T. Quartz-Seq: A Highly Reproducible and Sensitive Single-Cell RNA Sequencing Method, Reveals Non-Genetic Gene-Expression Heterogeneity. Genome Biol (2013) 14(4):R31. doi: 10.1186/gb-2013-14-4-r31 23594475 PMC4054835

[32] Kashima Y Sakamoto Y Kaneko K Seki M Suzuki Y Suzuki A. Single-Cell Sequencing Techniques From Individual to Multiomics Analyses. Exp Mol Med (2020) 52(9):1419–27. doi: 10.1038/s12276-020-00499-2 PMC8080663 32929221

[33] Habib N Avraham-Davidi I Basu A Burks T Shekhar K Hofree M. Massively Parallel Single-Nucleus RNA-Seq With DroNc-Seq. Nat Methods (2017) 14(10):955–8. doi: 10.1038/nmeth.4407 PMC5623139 28846088

[34] Han X Wang R Zhou Y Fei L Sun H Lai S. Mapping the Mouse Cell Atlas by Microwell-Seq. Cell (2018) 172(5):1091–107.e17. doi: 10.1016/j.cell.2018.02.001 29474909

[35] Hashimoto S. Nx1-Seq (Well Based Single-Cell Analysis System). Adv Exp Med Biol (2019) 1129:51–61. doi: 10.1007/978-981-13-6037-4_4 30968360

[36] Cao J Packer JS Ramani V Cusanovich DA Huynh C Daza R. Comprehensive Single-Cell Transcriptional Profiling of a Multicellular Organism. Science (2017) 357(6352):661–7. doi: 10.1126/science.aam8940 PMC5894354 28818938

[37] Cao J Spielmann M Qiu X Huang X Ibrahim DM Hill AJ. The Single-Cell Transcriptional Landscape of Mammalian Organogenesis. Nature (2019) 566(7745):496–502. doi: 10.1038/s41586-019-0969-x 30787437 PMC6434952

[38] Holmes M Zhang F Bieniasz PD. Single-Cell and Single-Cycle Analysis of HIV-1 Replication. PloS Pathog (2015) 11(6):e1004961. doi: 10.1371/journal.ppat.1004961 26086614 PMC4472667

[39] Takaori A. Antiviral Defense by APOBEC3 Family Proteins. Uirusu (2005) 55(2):267–72. doi: 10.2222/jsv.55.267 16557012

[40] Takaori-Kondo A. APOBEC Family Proteins: Novel Antiviral Innate Immunity. Int J Hematol (2006) 83(3):213–6. doi: 10.1532/ijh97.05187 16720550

[41] Wang S Zhang Q Hui H Agrawal K Karris MAY Rana TM. An Atlas of Immune Cell Exhaustion in HIV-Infected Individuals Revealed by Single-Cell Transcriptomics. Emerg Microbes Infect (2020) 9(1):2333–47. doi: 10.1080/22221751.2020.1826361 PMC7646563 32954948

[42] Gattinoni L Lugli E Ji Y Pos Z Paulos CM Quigley MF. A Human Memory T Cell Subset With Stem Cell-Like Properties. Nat Med (2011) 17(10):1290–7. doi: 10.1038/nm.2446 PMC3192229 21926977

[43] Farhadian SF Mehta SS Zografou C Robertson K Price RW Pappalardo J. Single-Cell RNA Sequencing Reveals Microglia-Like Cells in Cerebrospinal Fluid During Virologically Suppressed HIV. JCI Insight (2018) 3(18):e121718. doi: 10.1172/jci.insight.121718 PMC6237230 30232286

[44] Kazer SW Aicher TP Muema DM Carroll SL Ordovas-Montanes J Miao VN. Integrated Single-Cell Analysis of Multicellular Immune Dynamics During Hyperacute HIV-1 Infection. Nat Med (2020) 26(4):511–8. doi: 10.1038/s41591-020-0799-2 PMC7237067 32251406

[45] Baxter AE Niessl J Fromentin R Richard J Porichis F Charlebois R. Single-Cell Characterization of Viral Translation-Competent Reservoirs in HIV-Infected Individuals. Cell Host Microbe (2016) 20(3):368–80. doi: 10.1016/j.chom.2016.07.015 PMC5025389 27545045

[46] Nguyen S Deleage C Darko S Ransier A Truong DP Agarwal D. Elite Control of HIV Is Associated With Distinct Functional and Transcriptional Signatures in Lymphoid Tissue CD8(+) T Cells. Sci Transl Med (2019) 11(523):eaax4077. doi: 10.1126/scitranslmed.aax4077 31852798 PMC7265335

[47] Russell JH Ley TJ. Lymphocyte-Mediated Cytotoxicity. Annu Rev Immunol (2002) 20:323–70. doi: 10.1146/annurev.immunol.20.100201.131730 11861606

[48] Voskoboinik I Whisstock JC Trapani JA. Perforin and Granzymes: Function, Dysfunction and Human Pathology. Nat Rev Immunol (2015) 15(6):388–400. doi: 10.1038/nri3839 25998963

[49] Buggert M Tauriainen J Yamamoto T Frederiksen J Ivarsson MA Michaëlsson J. T-Bet and Eomes Are Differentially Linked to the Exhausted Phenotype of CD8+ T Cells in HIV Infection. PloS Pathog (2014) 10(7):e1004251. doi: 10.1371/journal.ppat.1004251 25032686 PMC4102564

[50] Wherry EJ Kurachi M. Molecular and Cellular Insights Into T Cell Exhaustion. Nat Rev Immunol (2015) 15(8):486–99. doi: 10.1038/nri3862 PMC4889009 26205583

[51] Tauriainen J Scharf L Frederiksen J Naji A Ljunggren HG Sönnerborg A. Perturbed CD8(+) T Cell TIGIT/CD226/PVR Axis Despite Early Initiation of Antiretroviral Treatment in HIV Infected Individuals. Sci Rep (2017) 7:40354. doi: 10.1038/srep40354 28084312 PMC5233961

[52] Carrette F Surh CD. IL-7 Signaling and CD127 Receptor Regulation in the Control of T Cell Homeostasis. Semin Immunol (2012) 24(3):209–17. doi: 10.1016/j.smim.2012.04.010 PMC3367861 22551764

[53] Ribeiro-Dias F Saar Gomes R de Lima Silva LL Dos Santos JC Joosten LA. Interleukin 32: A Novel Player in the Control of Infectious Diseases. J Leukoc Biol (2017) 101(1):39–52. doi: 10.1189/jlb.4RU0416-175RR 27793959

[54] Lane BR Markovitz DM Woodford NL Rochford R Strieter RM Coffey MJ. TNF-Alpha Inhibits HIV-1 Replication in Peripheral Blood Monocytes and Alveolar Macrophages by Inducing the Production of RANTES and Decreasing C-C Chemokine Receptor 5 (CCR5) Expression. J Immunol (1999) 163(7):3653–61. 10490959

[55] Scarlatti G Tresoldi E Björndal A Fredriksson R Colognesi C Deng HK. *In Vivo* Evolution of HIV-1 Co-Receptor Usage and Sensitivity to Chemokine-Mediated Suppression. Nat Med (1997) 3(11):1259–65. doi: 10.1038/nm1197-1259 9359702

[56] Appay V Rowland-Jones SL. RANTES: A Versatile and Controversial Chemokine. Trends Immunol (2001) 22(2):83–7. doi: 10.1016/s1471-4906(00)01812-3 11286708

[57] Bedoya VI Boasso A Hardy AW Rybak S Shearer GM Rugeles MT. Ribonucleases in HIV Type 1 Inhibition: Effect of Recombinant RNases on Infection of Primary T Cells and Immune Activation-Induced RNase Gene and Protein Expression. AIDS Res Hum Retroviruses (2006) 22(9):897–907. doi: 10.1089/aid.2006.22.897 16989616

[58] Zapata W Aguilar-Jiménez W Feng Z Weinberg A Russo A Potenza N. Identification of Innate Immune Antiretroviral Factors During *In Vivo* and *In Vitro* Exposure to HIV-1. Microbes Infect (2016) 18(3):211–9. doi: 10.1016/j.micinf.2015.10.009 26548606

[59] Monteleone K Di Maio P Cacciotti G Falasca F Fraulo M Falciano M. Interleukin-32 Isoforms: Expression, Interaction With Interferon-Regulated Genes and Clinical Significance in Chronically HIV-1-Infected Patients. Med Microbiol Immunol (2014) 203(3):207–16. doi: 10.1007/s00430-014-0329-2 24553842

[60] Zhao S Tsibris A. Leveraging Novel Integrated Single-Cell Analyses to Define HIV-1 Latency Reversal. Viruses (2021) 13(7):1197. doi: 10.3390/v13071197 34206546 PMC8310207

[61] Cohn LB da Silva IT Valieris R Huang AS Lorenzi JCC Cohen YZ. Clonal CD4(+) T Cells in the HIV-1 Latent Reservoir Display a Distinct Gene Profile Upon Reactivation. Nat Med (2018) 24(5):604–9. doi: 10.1038/s41591-018-0017-7 PMC5972543 29686423

[62] Liu R Yeh YJ Varabyou A Collora JA Sherrill-Mix S Talbot CC Jr.. Single-Cell Transcriptional Landscapes Reveal HIV-1-Driven Aberrant Host Gene Transcription as a Potential Therapeutic Target. Sci Transl Med (2020) 12(543):eaaz0802. doi: 10.1126/scitranslmed.aaz0802 32404504 PMC7453882

[63] Golumbeanu M Cristinelli S Rato S Munoz M Cavassini M Beerenwinkel N. Single-Cell RNA-Seq Reveals Transcriptional Heterogeneity in Latent and Reactivated HIV-Infected Cells. Cell Rep (2018) 23(4):942–50. doi: 10.1016/j.celrep.2018.03.102 29694901

[64] Mohammadi P di Iulio J Muñoz M Martinez R Bartha I Cavassini M. Dynamics of HIV Latency and Reactivation in a Primary CD4+ T Cell Model. PloS Pathog (2014) 10(5):e1004156. doi: 10.1371/journal.ppat.1004156 24875931 PMC4038609

[65] Zaongo SD Ma P Song FZ Chen YK. Selective Elimination of Host Cells Harboring Replication-Competent Human Immunodeficiency Virus Reservoirs: A Promising Therapeutic Strategy for HIV Cure. Chin Med J (2021) 134(23):2776–87. doi: 10.1097/cm9.0000000000001797 PMC8667983 34620750

[66] Szklarczyk D Morris JH Cook H Kuhn M Wyder S Simonovic M. The STRING Database in 2017: Quality-Controlled Protein-Protein Association Networks, Made Broadly Accessible. Nucleic Acids Res (2017) 45(D1):D362–d8. doi: 10.1093/nar/gkw937 PMC5210637 27924014

[67] Bradley T Ferrari G Haynes BF Margolis DM Browne EP. Single-Cell Analysis of Quiescent HIV Infection Reveals Host Transcriptional Profiles That Regulate Proviral Latency. Cell Rep (2018) 25(1):107–17.e3. doi: 10.1016/j.celrep.2018.09.020 30282021 PMC6258175

[68] Saito S Sakai M Sasaki Y Tanebe K Tsuda H Michimata T. Quantitative Analysis of Peripheral Blood Th0, Th1, Th2 and the Th1:Th2 Cell Ratio During Normal Human Pregnancy and Preeclampsia. Clin Exp Immunol (1999) 117(3):550–5. doi: 10.1046/j.1365-2249.1999.00997.x PMC1905376 10469061

[69] Bahbouhi B al-Harthi L. Enriching for HIV-Infected Cells Using Anti-Gp41 Antibodies Indirectly Conjugated to Magnetic Microbeads. BioTechniques (2004) 36(1):139–47. doi: 10.2144/04361rr05 14740496

[70] Yucha RW Hobbs KS Hanhauser E Hogan LE Nieves W Ozen MO. High-Throughput Characterization of HIV-1 Reservoir Reactivation Using a Single-Cell-In-Droplet PCR Assay. EBioMedicine (2017) 20:217–29. doi: 10.1016/j.ebiom.2017.05.006 PMC5478213 28529033

[71] Passaes CPB Bruel T Decalf J David A Angin M Monceaux V. Ultrasensitive HIV-1 P24 Assay Detects Single Infected Cells and Differences in Reservoir Induction by Latency Reversal Agents. J Virol (2017) 91(6):e02296–16. doi: 10.1128/jvi.02296-16 28077644 PMC5331803

[72] Abdel-Mohsen M Richman D Siliciano RF Nussenzweig MC Howell BJ Martinez-Picado J. Recommendations for Measuring HIV Reservoir Size in Cure-Directed Clinical Trials. Nat Med (2020) 26(9):1339–50. doi: 10.1038/s41591-020-1022-1 PMC7703694 32895573

[73] Raghuvanshi R Bharate SB. Preclinical and Clinical Studies on Bryostatins, A Class of Marine-Derived Protein Kinase C Modulators: A Mini-Review. Curr Top Med Chem (2020) 20(12):1124–35. doi: 10.2174/1568026620666200325110444 32209043

[74] Chomont N El-Far M Ancuta P Trautmann L Procopio FA Yassine-Diab B. HIV Reservoir Size and Persistence Are Driven by T Cell Survival and Homeostatic Proliferation. Nat Med (2009) 15(8):893–900. doi: 10.1038/nm.1972 19543283 PMC2859814

[75] Sannier G Dubé M Dufour C Richard C Brassard N Delgado GG. Combined Single-Cell Transcriptional, Translational, and Genomic Profiling Reveals HIV-1 Reservoir Diversity. Cell Rep (2021) 36(9):109643. doi: 10.1016/j.celrep.2021.109643 34469719

[76] Hiener B Horsburgh BA Eden JS Barton K Schlub TE Lee E. Identification of Genetically Intact HIV-1 Proviruses in Specific CD4(+) T Cells From Effectively Treated Participants. Cell Rep (2017) 21(3):813–22. doi: 10.1016/j.celrep.2017.09.081 PMC5960642 29045846

[77] Foster JL Garcia JV. Role of Nef in HIV-1 Replication and Pathogenesis. Adv Pharmacol (2007) 55:389–409. doi: 10.1016/s1054-3589(07)55011-8 17586321

[78] Lu CL Pai JA Nogueira L Mendoza P Gruell H Oliveira TY. Relationship Between Intact HIV-1 Proviruses in Circulating CD4(+) T Cells and Rebound Viruses Emerging During Treatment Interruption. Proc Natl Acad Sci USA (2018) 115(48):E11341–8. doi: 10.1073/pnas.1813512115 PMC6275529 30420517

[79] Bruner KM Murray AJ Pollack RA Soliman MG Laskey SB Capoferri AA. Defective Proviruses Rapidly Accumulate During Acute HIV-1 Infection. Nat Med (2016) 22(9):1043–9. doi: 10.1038/nm.4156 PMC5014606 27500724

[80] Bruner KM Wang Z Simonetti FR Bender AM Kwon KJ Sengupta S. A Quantitative Approach for Measuring the Reservoir of Latent HIV-1 Proviruses. Nature (2019) 566(7742):120–5. doi: 10.1038/s41586-019-0898-8 PMC6447073 30700913

[81] Ho YC Shan L Hosmane NN Wang J Laskey SB Rosenbloom DI. Replication-Competent Noninduced Proviruses in the Latent Reservoir Increase Barrier to HIV-1 Cure. Cell (2013) 155(3):540–51. doi: 10.1016/j.cell.2013.09.020 PMC3896327 24243014

[82] Lee GQ Orlova-Fink N Einkauf K Chowdhury FZ Sun X Harrington S. Clonal Expansion of Genome-Intact HIV-1 in Functionally Polarized Th1 CD4+ T Cells. J Clin Investig (2017) 127(7):2689–96. doi: 10.1172/jci93289 PMC5490740 28628034

[83] Barouch DH Deeks SG. Immunologic Strategies for HIV-1 Remission and Eradication. Science (2014) 345(6193):169–74. doi: 10.1126/science.1255512 PMC4096716 25013067

[84] Cheng L Yu H Wrobel JA Li G Liu P Hu Z. Identification of Pathogenic TRAIL-Expressing Innate Immune Cells During HIV-1 Infection in Humanized Mice by scRNA-Seq. JCI Insight (2020) 5(11):e135344. doi: 10.1172/jci.insight.135344 PMC7308046 32406872

[85] Cummins N Badley A. The TRAIL to Viral Pathogenesis: The Good, the Bad and the Ugly. Curr Mol Med (2009) 9(4):495–505. doi: 10.2174/156652409788167078 19519406 PMC3149795

[86] Gandhi RT Chen BK Straus SE Dale JK Lenardo MJ Baltimore D. HIV-1 Directly Kills CD4+ T Cells by a Fas-Independent Mechanism. J Exp Med (1998) 187(7):1113–22. doi: 10.1084/jem.187.7.1113 PMC2212217 9529327

[87] Mongkolsapaya J Cowper AE Xu XN Morris G McMichael AJ Bell JI. Lymphocyte Inhibitor of TRAIL (TNF-Related Apoptosis-Inducing Ligand): A New Receptor Protecting Lymphocytes From the Death Ligand TRAIL. J Immunol (1998) 160(1):3–6. 9551946

[88] Screaton GR Mongkolsapaya J Xu XN Cowper AE McMichael AJ Bell JI. TRICK2, A New Alternatively Spliced Receptor That Transduces the Cytotoxic Signal From TRAIL. Curr Biol (1997) 7(9):693–6. doi: 10.1016/s0960-9822(06)00297-1 9285725

[89] Tabb B Morcock DR Trubey CM Quiñones OA Hao XP Smedley J. Reduced Inflammation and Lymphoid Tissue Immunopathology in Rhesus Macaques Receiving Anti-Tumor Necrosis Factor Treatment During Primary Simian Immunodeficiency Virus Infection. J Infect Dis (2013) 207(6):880–92. doi: 10.1093/infdis/jis643 PMC3571439 23087435

[90] Niu M Morsey B Lamberty BG Emanuel K Yu F León-Rivera R. Methamphetamine Increases the Proportion of SIV-Infected Microglia/Macrophages, Alters Metabolic Pathways, and Elevates Cell Death Pathways: A Single-Cell Analysis. Viruses (2020) 12(11):1297. doi: 10.3390/v12111297 PMC7697917 33198269

[91] UNAIDS. AIDSinfo (2020). Available at: http://aidsinfo.unaids.org/ (Accessed December 3, 2021).

[92] Spach DH. Antiretroviral Medications and Initial Therapy (2021). Available at: https://www.hiv.uw.edu/go/antiretroviral-therapy/general-information/core-concept/all (Accessed December 3, 2021).

[93] Volberding PA Deeks SG. Antiretroviral Therapy and Management of HIV Infection. Lancet (2010) 376(9734):49–62. doi: 10.1016/S0140-6736(10)60676-9 20609987

[94] Kalichman SC Cherry C Amaral CM Swetzes C Eaton L Macy R. Adherence to Antiretroviral Therapy and HIV Transmission Risks: Implications for Test-and-Treat Approaches to HIV Prevention. AIDS Patient Care STDs (2010) 24(5):271–7. doi: 10.1089/apc.2009.0309 PMC2875951 20438373

[95] Wilson EM Sereti I. Immune Restoration After Antiretroviral Therapy: The Pitfalls of Hasty or Incomplete Repairs. Immunol Rev (2013) 254(1):343–54. doi: 10.1111/imr.12064 PMC3694599 23772630

[96] Autran B Carcelain G Li TS Blanc C Mathez D Tubiana R. Positive Effects of Combined Antiretroviral Therapy on CD4+ T Cell Homeostasis and Function in Advanced HIV Disease. Science (1997) 277(5322):112–6. doi: 10.1126/science.277.5322.112 9204894

[97] Michaels SH Clark R Kissinger P. Declining Morbidity and Mortality Among Patients With Advanced Human Immunodeficiency Virus Infection. N Engl J Med (1998) 339(6):405–6. doi: 10.1056/nejm199808063390612 9696654

[98] Le Guillou A Buchbinder S Scott H Liu A Havlir D Scheer S. Population Impact and Efficiency of Improvements to HIV PrEP Under Conditions of High ART Coverage Among San Francisco Men Who Have Sex With Men. J Acquir Immune Defic Syndr (2021) 88(4):340–7. doi: 10.1097/qai.0000000000002781 PMC8556308 34354011

[99] Cohen MS Chen YQ McCauley M Gamble T Hosseinipour MC Kumarasamy N. Prevention of HIV-1 Infection With Early Antiretroviral Therapy. N Engl J Med (2011) 365(6):493–505. doi: 10.1056/NEJMoa1105243 21767103 PMC3200068

[100] Kelley CF Kitchen CM Hunt PW Rodriguez B Hecht FM Kitahata M. Incomplete Peripheral CD4+ Cell Count Restoration in HIV-Infected Patients Receiving Long-Term Antiretroviral Treatment. Clin Infect Dis (2009) 48(6):787–94. doi: 10.1086/597093 PMC2720023 19193107

[101] Rocafort M Noguera-Julian M Rivera J Pastor L Guillén Y Langhorst J. Evolution of the Gut Microbiome Following Acute HIV-1 Infection. Microbiome (2019) 7(1):73. doi: 10.1186/s40168-019-0687-5 31078141 PMC6511141

[102] Klatt NR Funderburg NT Brenchley JM. Microbial Translocation, Immune Activation, and HIV Disease. Trends Microbiol (2013) 21(1):6–13. doi: 10.1016/j.tim.2012.09.001 23062765 PMC3534808

[103] Sessa L Reddel S Manno E Quagliariello A Cotugno N Del Chierico F. Distinct Gut Microbiota Profile in Antiretroviral Therapy-Treated Perinatally HIV-Infected Patients Associated With Cardiac and Inflammatory Biomarkers. AIDS (2019) 33(6):1001–11. doi: 10.1097/qad.0000000000002131 30946154

[104] Lu W Feng Y Jing F Han Y Lyu N Liu F. Association Between Gut Microbiota and CD4 Recovery in HIV-1 Infected Patients. Front Microbiol (2018) 9:1451. doi: 10.3389/fmicb.2018.01451 30034377 PMC6043814

[105] Nowak P Troseid M Avershina E Barqasho B Neogi U Holm K. Gut Microbiota Diversity Predicts Immune Status in HIV-1 Infection. AIDS (2015) 29(18):2409–18. doi: 10.1097/qad.0000000000000869 26355675

[106] Vesterbacka J Rivera J Noyan K Parera M Neogi U Calle M. Richer Gut Microbiota With Distinct Metabolic Profile in HIV Infected Elite Controllers. Sci Rep (2017) 7(1):6269. doi: 10.1038/s41598-017-06675-1 28740260 PMC5524949

[107] Boasso A Shearer GM Chougnet C. Immune Dysregulation in Human Immunodeficiency Virus Infection: Know It, Fix It, Prevent It? J Intern Med (2009) 265(1):78–96. doi: 10.1111/j.1365-2796.2008.02043.x 19093962 PMC2903738

[108] BL MR Drouin O Bartlett G Nguyen Q Low A Gavriilidis G. Incidence and Prevalence of Opportunistic and Other Infections and the Impact of Antiretroviral Therapy Among HIV-Infected Children in Low- and Middle-Income Countries: A Systematic Review and Meta-Analysis. Clin Infect Dis (2016) 62(12):1586–94. doi: 10.1093/cid/ciw139 PMC4885647 27001796

[109] Low A Gavriilidis G Larke N MR BL Drouin O Stover J. Incidence of Opportunistic Infections and the Impact of Antiretroviral Therapy Among HIV-Infected Adults in Low- and Middle-Income Countries: A Systematic Review and Meta-Analysis. Clin Infect Dis (2016) 62(12):1595–603. doi: 10.1093/cid/ciw125 PMC4885646 26951573

[110] Prasitsuebsai W Kariminia A Puthanakit T Lumbiganon P Hansudewechakul R Siew Moy F. Impact of Antiretroviral Therapy on Opportunistic Infections of HIV-Infected Children in the Therapeutic Research, Education and AIDS Training Asia Pediatric HIV Observational Database. Pediatr Infect Dis J (2014) 33(7):747–52. doi: 10.1097/inf.0000000000000226 PMC4055535 24378942

